# Yeast hydrolysate attenuates lipopolysaccharide-induced inflammatory responses and intestinal barrier damage in weaned piglets

**DOI:** 10.1186/s40104-023-00835-2

**Published:** 2023-03-17

**Authors:** Runqi Fu, Chan Liang, Daiwen Chen, Gang Tian, Ping Zheng, Jun He, Jie Yu, Xiangbing Mao, Yuheng Luo, Junqiu Luo, Bing Yu

**Affiliations:** 1 Key Laboratory of Animal Disease-Resistance Nutrition, Ministry of Education, Ministry of Agriculture and Rural Affairs, Key Laboratory of Sichuan Province, Chengdu, 611130 China; 2 Institute of Animal Nutrition, Sichuan Agricultural University, Chengdu, 611130 Sichuan China

**Keywords:** Inflammatory response, Intestinal barrier, Lipopolysaccharide, Piglets, Yeast hydrolysate

## Abstract

**Background:**

Intestinal inflammation is the main risk factor causing intestinal barrier dysfunction and lipopolysaccharide (LPS) can trigger inflammatory responses in various eukaryotic species. Yeast hydrolysate (YH) possesses multi-biological effects and is received remarkable attention as a functional ingredient for improving growth performance and promoting health in animals. However, there is still inconclusive on the protective effects of dietary YH supplementation on intestinal barrier of piglets. This study was conducted to investigate the attenuate effects of YH supplementation on inflammatory responses and intestinal barrier injury in piglets challenged with LPS.

**Methods:**

Twenty-four piglets (with an average body weight of 7.42 ± 0.34 kg) weaned at 21 days of age were randomly assigned to one of two dietary treatments (12 replications with one pig per pen): a basal diet or a basal diet containing YH (5 g/kg). On the 22^nd^ d, 6 piglets in each treatment were intraperitoneally injected with LPS at 150 μg/kg BW, and the others were injected with the same amount of sterile normal saline. Four hours later, blood samples of each piglet were collected and then piglets were euthanized.

**Results:**

Dietary YH supplementation increased average daily feed intake and average daily gain (*P* < 0.01), decreased the ratio of feed intake to gain of piglets (*P* = 0.048). Lipopolysaccharide (LPS) injection induced systemic inflammatory response, evidenced by the increase of serum concentrations of haptoglobin (HP), adrenocorticotropic hormone (ACTH), cortisol, and interleukin-1β (IL-1β). Furthermore, LPS challenge resulted in inflammatory intestinal damage, by up-regulation of the protein or mRNA abundances of tumor necrosis factor-*α* (TNF-α), IL-1β, toll-like receptors 4 (TLR4) and phosphor-nuclear factor-κB-p65 (p-NFκB-p65) (*P* < 0.01), and down-regulation of the jejunal villus height, the protein and mRNA abundances of zonula occludens-1 (ZO-1) and occludin (OCC; *P* < 0.05) in jejunal mucosa. Dietary YH supplementation decreased the impaired effects of ACTH, cortisol, HP, IL-1β and diamine oxidase in serum (*P* < 0.05). Moreover, YH supplementation also up-regulated the jejunal villus height, protein and mRNA abundances of ZO-1 and OCC (*P* < 0.05), down-regulated the mRNA expressions of *TNF-α* and *IL-1β* and the protein abundances of TNF-α, IL-1β, TLR4 and p-NFκB-p65 in jejunal mucosa in LPS-challenged pigs (*P* < 0.01).

**Conclusion:**

Yeast hydrolysate could attenuate inflammatory response and intestinal barrier injury in weaned piglets challenged with LPS, which was associated with the inhibition of TLR4/NF-κB signaling pathway activation.

**Graphical Abstract:**

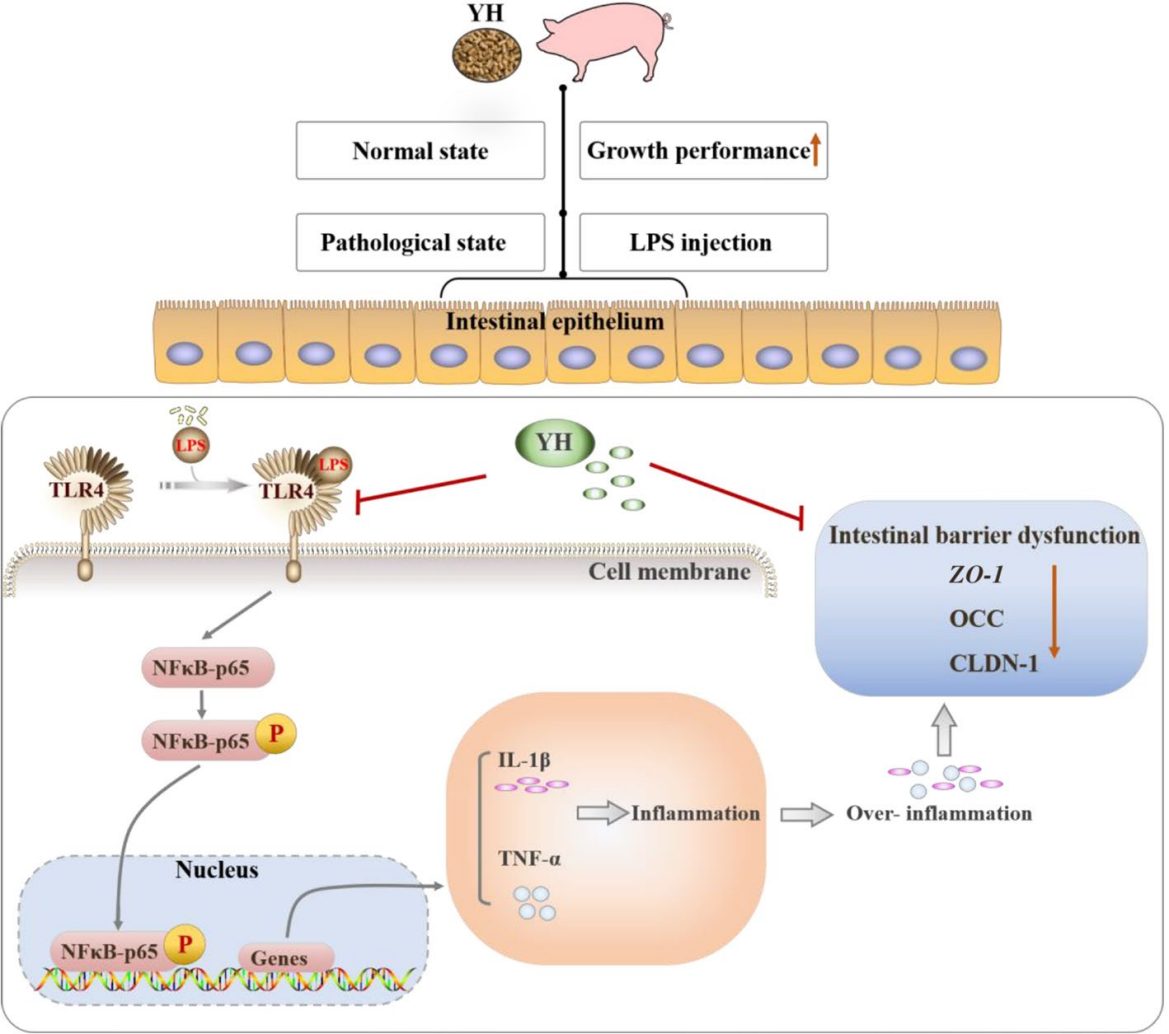

**Supplementary Information:**

The online version contains supplementary material available at 10.1186/s40104-023-00835-2.

## Introduction

Intestinal epithelial barrier, a structure of continuous monolayer enterocytes, is dominated by intercellular tight junction [1]. Under a normal state, it acts as a selective filter that enables the absorption of nutrients and ensures an effective defense against exogenous pathogens, luminal antigens, etc. [2]. For early weaned mammals, however, the gastrointestinal tract is immature and vulnerable to multitudinous stresses [3], and intestinal epithelial barrier is frequently defective in various pathological status, especially in bacterial infections induced by pathogenic bacteria [4, 5]. The damaged intestinal epithelial barrier mainly causes the increase of intestinal permeability, promotes the transfer of antigens in lumen to the subepithelial tissues, and further exacerbates the mucosal and systemic inflammatory reactions [1, 6, 7]. The secretion of pro-inflammatory cytokines during the inflammatory response is a major factor in triggering the disruption of the intestinal barrier [8]. In piglets, intestinal barrier dysfunction and accompanied by the enhancement of intestinal permeability are often observed, with subsequent diarrhea and growth retardation [9, 10]. Therefore, effective and safe preventive approaches to maintenance of intestinal barrier function are urgently needed for piglets. Notably, lipopolysaccharide (LPS), an intrinsic component of the cell wall of gram-negative bacteria, has been shown to be a key molecule in inducing the production of pro-inflammatory cytokines [7]. It often contributes to inflammatory intestinal damage in piglets. The molecular mechanism is manifested by the activation of inflammatory signaling pathway by LPS, which induces the expression of key proteins of pro-inflammatory factors and thus leads to intestinal barrier damage [11]. Previous studies have shown that LPS can be used to construct a well-established model of inflammation in pigs [12, 13].

Yeast (*Saccharomyces cerevisiae*) is widely distributed in nature and has been inseparable from human life [14]. Yeast hydrolysate (YH), also known as yeast autolysate, is obtained from *Saccharomyces cerevisiae* via protein hydrolysis enzyme [15, 16]. Several researches suggested that the autolytic yeast fractions or peptides from autolyzed yeasts revealed physiological effects on anti-obesity [17, 18], anti-fatigue [19], anti-stress [20, 21] and immuno-promotional activities [22, 23]. For these reasons, YH has attracted much attention as a functional material supplement and it is generally recognized as non-toxic, effective and safe [24]. Recently, several studies were focused on improving intestinal health and immune-potentiating activities with YH. Specifically, YH has multiple roles on promoting digestion and absorption of nutrients [25], improving intestinal microflora structure [26] and decreasing diarrhea of young animals [19], while also acts as an immunomodifier to prevent gut inflammation [27]. However, the protective effects of dietary supplementation with YH on intestinal barrier are limited and inconclusive. It is widely known that hyperinflammation in intestine is one of the most factors causing intestinal barrier dysfunction [1, 2, 10]. Consequently, considering the above, we postulated that YH has the potential to prevent LPS-induced intestinal barrier dysfunction in piglets by alleviating inflammation via the related signaling pathways. This study tested these hypotheses by assessing the effects of YH on the systemic inflammatory response, intestinal morphology, expression of tight junction-related proteins and gut anti-inflammatory capacity in piglets.

## Materials and methods

### Chemical analysis of yeast hydrolysate

Yeast hydrolysate was provided by Jiangmen Thealth Bioengineering Co., Ltd. (Guangzhou, China). The contents of moisture, crude protein, crude fat and crude ash were measured with the reference of AOAC [28]. Gross energy was determined by an oxygen bomb calorimeter (Parr instruments, Moline, IL, USA). The soluble protein in yeast hydrolysate was extracted according to the method of Wang et al. [29] and fractionated by sodium dodecyl sulfate–polyacrylamide gel electrophoresis (SDS-PAGE) system according to the previous study [30]. The gel was stained with Coomassie Brilliant Blue (Beyotime, Shanghai, China) for 40 min and de-stained with deionized water for 8 h.

### Experimental animals, diet, and design

**Fig. 1:**
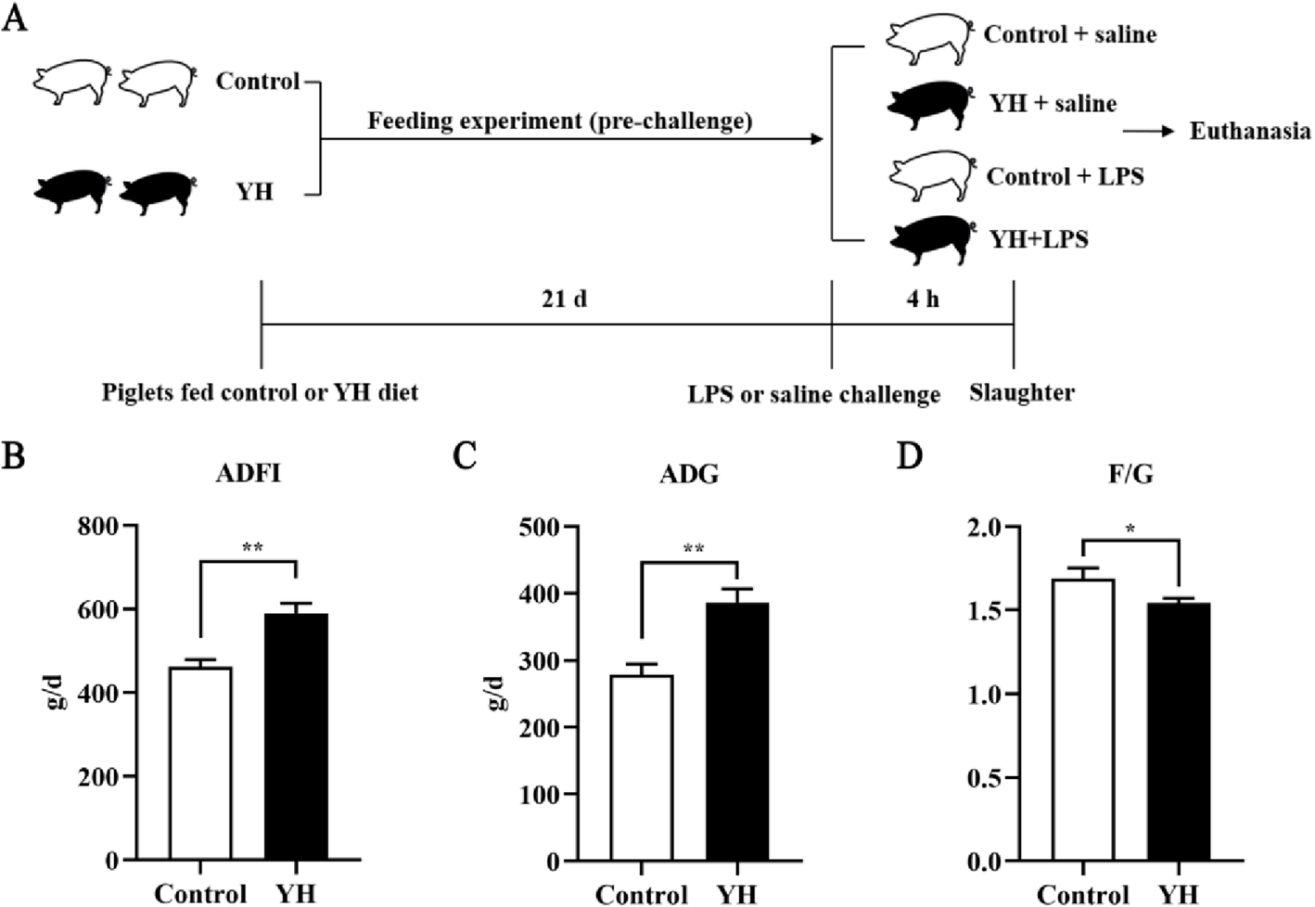
Dietary yeast hydrolysate supplementation improved the growth performance of weaned piglets before LPS challenge. **A** Schematic of the feeding experiment and LPS challenge. **B–D** Effects of dietary YH supplementation on average daily feed intake (ADFI), average daily gain (ADG) and the ratio of feed intake to gain (F/G) of weaned piglets. Control, piglets were fed with a basal diet; YH, piglets were fed with a YH containing diet, 5 g/kg. ^*^P < 0.05, ^**^P < 0.01, compared with control group

The animal procedures were reviewed and approved by the Animal Care and Use Committee at Sichuan Agricultural University with the approval number of SYXK (Sichuan)-2019–187. A total of twenty-four weaned piglets (21-day-old), with an average initial body weight (BW) of 7.42 ± 0.34 kg, were randomly allotted to two groups (12 replicates per treatment and one piglets per replicate) receiving either a basal diet or a basal diet with YH (5 g/kg) for 21 d. All piglets were fed individually in metabolic cages (1.5 m × 0.7 m × 1.0 m) and housed in an environmentally controlled room. Piglets were fed three times daily at 8:00, 14:00 and 20:00 and have free accessed to water and feed throughout the experiment. After 21-d feeding trial, immunological challenge was applied to the half of piglets in each treatment (Fig. 1A). That means 6 piglets in each treatment were intraperitoneally injected with LPS at 150 μg/kg BW, and the other 6 piglets were injected with an equal volume of sterile physiological saline. The basal diet (Table 1) was a corn-soybean meal-fish meal diet and was formulated to meet or exceed National Research Council (NRC 2012) [31] requirements for piglets from 7–11 kg and 11–25 kg stage. The YH diet was formulated by replacing soybean meal with 5 g/kg YH in equal amounts in the basal diet. The molecular weight of YH was less than 50 kDa and mostly clustered below 25 kDa (Fig. S1). YH mainly provided a rich source of crude protein (45.50%), and contributed less to crude ash (6.47%) and crude fat (2.17%) (Table S1).

**Table 1:** Composition and nutrients levels of the basal diet (air-dry basis, %)

**Ingredients**	**Content, %**	**Nutrient level**^3^	Content
Corn	31.42	Digestible energy, MJ/kg	14.73
Extruded corn	30.00	Crude protein, %	18.50
Soybean meal	9.94	Ca, %	0.75
Extruded soybean	8.00	Total P, %	0.56
Fish meal	4.00	Available P, %	0.37
Whey powder	5.00	Digestible Lys, %	1.30
Soybean protein concentrate	5.00	Digestible Met, %	0.38
Soybean oil	1.90	Digestible Thr, %	0.77
Sucrose	2.00	Digestible Trp, %	0.21
Limestone	0.85		
Dicalcium phosphate	0.45		
NaCl	0.20		
*L*-Lys·HCl (78%)	0.33		
*DL-*Met (99%)	0.07		
*L-*Thr (98.5%)	0.03		
*L-*Trp (98%)	0.01		
Chloride choline	0.15		
Vitamin premix^1^	0.05		
Mineral premix^2^	0.30		
Benzoic acid	0.30		
Total	100.00		

^1^Vitamin premix provided the following per kg of diet: VA 9000 IU, VD_3_ 3000 IU, VE 20.0 IU, VK_3_ 3.0 mg, VB_1_ 1.5 mg, VB_2_ 4.0 mg, VB_6_ 3.0 mg, VB_12_ 0.02 mg, nicotinic acid 30.0 mg, pantothenic acid 15.0 mg, folic acid 0.75 mg, biotin 0.1 mg

^2^Mineral premix provided the following per kg of diet: Fe (FeSO_4_·H_2_O) 100.0 mg, Cu (CuSO_4_·5H_2_O) 6.0 mg, Zn (ZnSO_4_·H_2_O) 100.0 mg, Mn (MnSO_4_·H_2_O) 4.0 mg, I (KI) 0.14 mg, Se (Na_2_SeO_3_) 0.3 mg

^3^Nutrients levels were calculated values

### LPS injection

The challenged piglets were intraperitoneally injected with *Escherichia coli* LPS (*E. coli* serotype O55:B5, Sigma Chemical Inc., St. Louis, MO, USA) at 150 μg/kg BW, and unchallenged piglets were administrated the same volume of sterile physiological saline. The dose and serotype of LPS used in this study was consistent with the previous reports [12, 13]. Previous experiments have presented that LPS injection particularly caused dramatic inflammatory response and intestinal barrier dysfunction in pigs, rats and mice. And these negative effects generally occurred within 3–6 h after LPS injection [7, 32]. Therefore, blood and intestinal samples in this study were collected 4 h following LPS or saline injection.

### Growth performance

Yeast hydrolysate treatment was a main factor prior to the LPS challenge. Piglets were weighted individually on 1^st^ d and 22^nd^ d of the experiment. Daily feed consumption was recorded for each piglet. Average daily gain and the ratio of feed intake to gain were calculated as well.

### Blood sample collection and analysis

Four hours following LPS and saline injection, blood samples were collected in 10 mL vacutainer tubes via anterior vena cava. Blood was centrifugated at 3500 × *g* for 10 min at 4 ℃. Serum samples were stored at −20 ℃ until subsequent analysis for inflammatory markers and diamine oxidase (DAO) concentrations. Commercially available porcine ELISA kits (Chenglin Biological Technology Co., Ltd., Beijing, China) were performed according to the manufacturer’s instructions for the following indicators: adrenocorticotropic hormone (ACTH), cortisol, C-reactive protein (CRP), serum amyloid A (SAA), haptoglobin (HP), tumor necrosis factor-α (TNF-α), interleukin-1β (IL-1β) and DAO in serum.

### Intestinal samples collection and analysis

Piglets were euthanized with pentobarbital sodium (200 mg/kg) in a separate sampling room away from other animals. The intestine was immediately removed. A 2-cm segment was removed from mid-jejunum and fixed with 4% paraformaldehyde solution. Paraffin embedding was used to cut into cross sects (5 μm thick). The jejunal morphology was determined by hematoxylin and eosin (H&E) stain. Intestinal morphological images were photographed with a Nikon TS100 microscope (40 × and 100 ×). Villus height and crypt depth were analyzed and calculated by Image Pro Plus 6.0 software (Media Cybernetics, Bethesda, MD, USA). Other sections were stained using immunofluorescence for TLR4 protein. Briefly, mouse anti-TLR4 monoclonal antibody (1:100, sc-293072, Santa Cruz, Dallas, TX, USA) was incubated overnight at 4 ℃. Corresponding secondary antibody (Cy3 conjugated Goat Anti-mouse IgG, 1:300, GB21301 from Servicebio, Wuhan, China) was incubated for 50 min at room temperature. The slides were washed three times with PBS, and then incubated with DAPI solution at room temperature for 10 min and stored in the dark. After immunofluorescence, microphotographs were acquired with an inverted microscope (Leica DMI400B, Wetzlar, Germany). In addition, the inner wall of the middle jejunum was washed with ice-cold saline and the mucosal samples were then scraped into a sterile tube. Mucosal samples were immediately placed into liquid nitrogen and stored at −80℃ until the analysis of genes and proteins expressions. About 0.5 g of frozen jejunal mucosal scrapings were homogenized in ice-cold saline and prepared into a 10% homogenate, crushed using an ultrasonic cell crushing system at 4 °C and then centrifuged (3000 × *g*, 15 min, 4 °C). The collected supernatant was used to analyze TNF-α and IL-1β contents by ELISA kits according to the manufacturer’s instructions.

### mRNA abundance analysis

Total RNA was extracted from jejunal mucosa using the TRIZOL reagent (TaKaRa Biotechnology (Dalian) Co., Ltd., Dalian, China). RNA integrity was verified by agarose gel electrophoresis. cDNA was synthesized with PrimeScript RT kit (TaKaRa). Real-time PCR was performed using SYBR Premix Ex Taq reagents (TaKaRa) and CFX-96 RT-qPCR Detection System (Bio-Rad, Hercules, CA, USA). The genes of intestinal barrier and inflammatory markers related primer pairs were synthesized by Sangon Biotech (Shanghai) Co., Ltd. (Shanghai, China) and listed in Table 2. The mRNA expression of target gene relative to housekeeping gene (*β-actin*) was calculated by the method of Arce et al. [33].

**Table 2:** Primer sequences used for real-time PCR

Gene	Primer sequence (5’ →3’)	Product length, bp	Accession number
*β-actin*	F: TCTGGCACCACACCTTCT	114	DQ178122
	R: TGATCTGGGTCATCTTCTCAC		
*ZO-1*	F: CAGCCCCCGTACATGGAGA	114	XM_005659811
	R: GCGCAGACGGTGTTCATAGTT		
*OCC*	F: CTACTCGTCCAACGGGAAAG	158	NM_001163647.2
	R: ACGCCTCCAAGTTACCACTG		
*ClDN-1*	F: TCTTAGTTGCCACAGCATGG	106	NM001244539
	R: CCAGTGAAGAGAGCCTGACC		
*MUC2*	F: GGTCATGCTGGAGCTGGACAGT	181	XM_003122394.1
	R: TGCCTCCTCGGGGTCGTCAC		
*IL-1β*	F: CAGCTGCAAATCTCTCACCA	112	NM_214055.1
	R: TCTTCATCGGCTTCTCCACT		
*TNF-α*	F: CGTGAAGCTGAAAGACAACCAG	121	NM_214022.1
	R: GATGGTGTGAGTGAGGAAAACG		
	R: CAGGCTTCCGTCATCTGGTT		

*CLDN-1* Claudin-1, *OCC* Occludin, *MUC2* Mucin2, *IL-1β* interleukin-1β, *TNF-α* Tumor necrosis factor-α, *ZO-1* Zonula occludens-1

### Western blot analysis

Western blot analysis was performed as previously described [34]. Briefly, protein was extracted from jejunal mucosa using the lysis buffer (Beyotime, Shanghai, China). Protein concentration was measured with the BCA protein assay kit (Pierce, Rockford, IL, USA). Then, protein was transferred to polyvinylidene fluoride membranes using a wet Trans-Blot system (Bio-Rad). After blocking, membranes were incubated with primary antibodies: anti-TLR4 (sc-293072, Santa Cruz), anti-ZO-1 (61–7300, Invitrogen, MA, USA), anti-OCC (ab31721, abcam, Shanghai, China), anti-TNF-α (ab6671, abcam), anti-IL-1β (sc-12742, Santa Cruz), anti-NFκB-p65 (6956, CST, Cell signaling Technology, Beverly, USA), anti-p-NFκB-p65 (3033, CST), and anti-β-actin (sc-47778, Santa Cruz). After washing, the corresponding secondary antibodies, goat anti-rabbit/mouse IgG -HRP secondary antibody (sc-2030 and sc-2031, Santa Cruz), were incubated at room temperature for 1 h. Visualization of membranes was performed with the Clarity™ Western ECL substrate (Bio-Rad) and the ChemiDoc XRS imaging system (Bio-Rad). The β-actin was applied as a controller for the mean of protein load.

### Statistical analysis

Statistical analysis was performed using SAS software package (Version 9.4; S.A.S, Institute Inc., Cary, NC, USA) [35]. All data were expressed as mean values with their standard error and checked for normal distribution using the Shapiro–Wilk test of SAS. Each piglet served as the statistical unit. Specifically, data on growth performance prior to LPS challenge were analyzed by two-tailed Student’s *t*-test. After LPS injection, data from serum and jejunum samples were statistically analyzed by two-way ANOVA using the PROC MIXED procedure of SAS with the following model:

$${y}_{ijk} = \mu + {\alpha }_{i} + {\beta }_{j} + {\alpha }_{i} \times {\beta }_{j} + {e}_{ijk}$$

where *y*_*ij*_ is an observed trait, *μ* is the overall mean, *α*_*i*_ is the fixed effect of immunological challenge (*i* = saline or LPS), *β*_*j*_ is the fixed effect of dietary YH (*j* = 0 or 5 g/kg YH), *α*_*i*_ × *β*_*j*_ is the interaction between LPS and YH and *e*_*ijk*_ is the random error. Differences between the different groups were analyzed by Duncan’s multiple comparison method. *P* < 0.05 was considered statistically significant, and 0.05 < *P* < 0.10 indicated a trend.

## Results

### Effects of YH on growth performance in piglets prior to LPS injection

As shown in Fig. 1, a 21-d feeding experiment was conducted to examine the effects of YH on growth performance of piglets under normal condition (Fig. 1A). Compared with control group, dietary YH supplementation increased ADFI (*P* < 0.01) and ADG (*P* < 0.01) (Fig. 1B and C), decreased F/G of piglets (*P* = 0.048) (Fig. 1D).

### Effects of YH on systemic inflammatory response and serum DAO concentration in piglets challenged with LPS

**Fig. 2:**
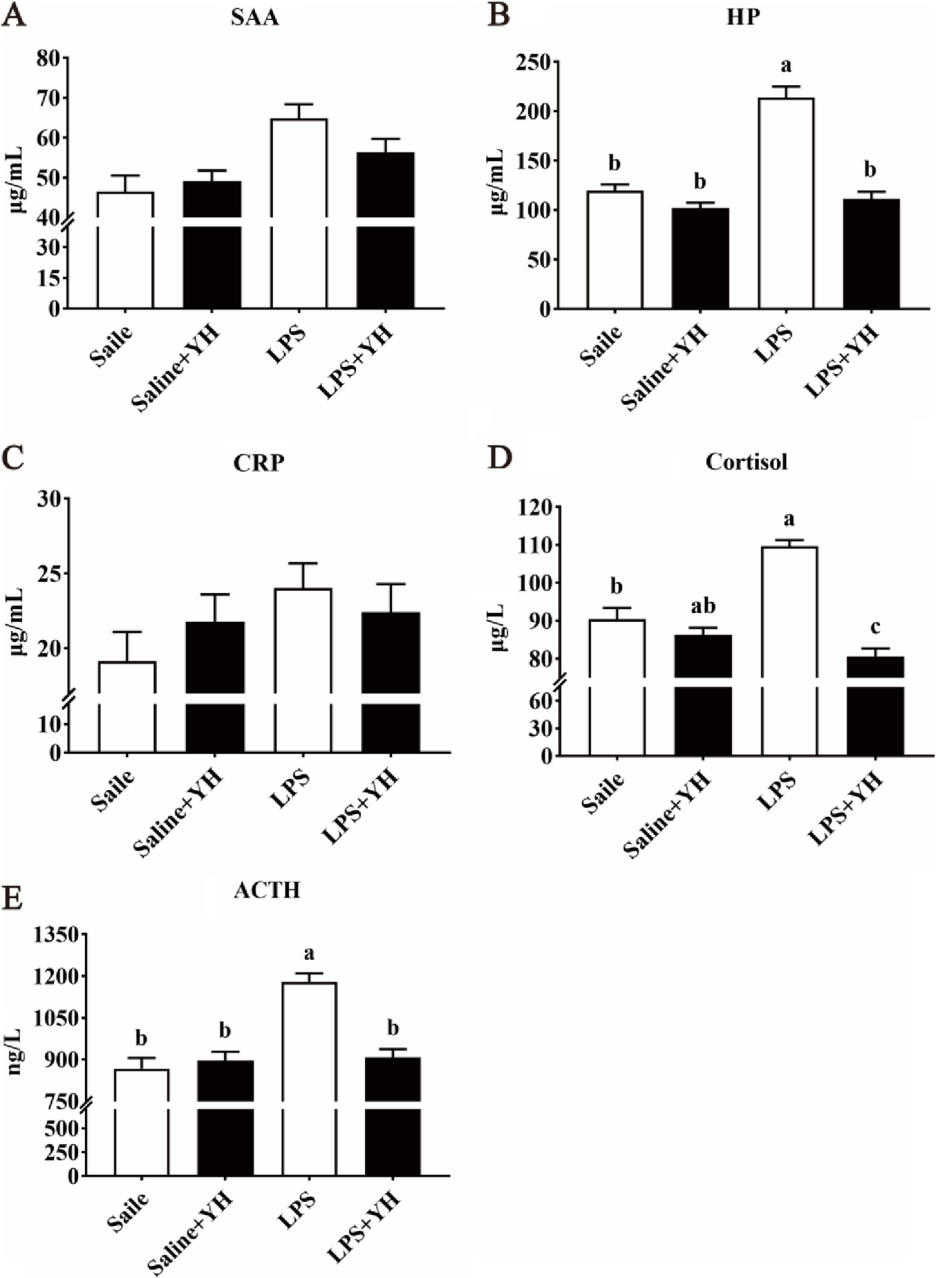
Dietary yeast hydrolysate supplementation inhibited the over-production of acute phase protein and stress hormone in piglets challenged with LPS. **A–C** Effects of dietary YH supplementation on the serum concentrations of serum amyloid A (SAA), haptoglobin (HP) and C-reactive protein (CRP) in piglets challenged with LPS. **D** and **E** Effects of dietary YH supplementation on the serum levels of stress hormones (**D)** cortisol and (**E)** adrenocorticotropic hormone. Control, piglets were fed with a basal diet; YH, piglets were fed with a YH containing diet, 5 g/kg. ^a,b,c^Means with different superscript letters in a row were significantly different (*P* < 0.05)

**Fig. 3:**
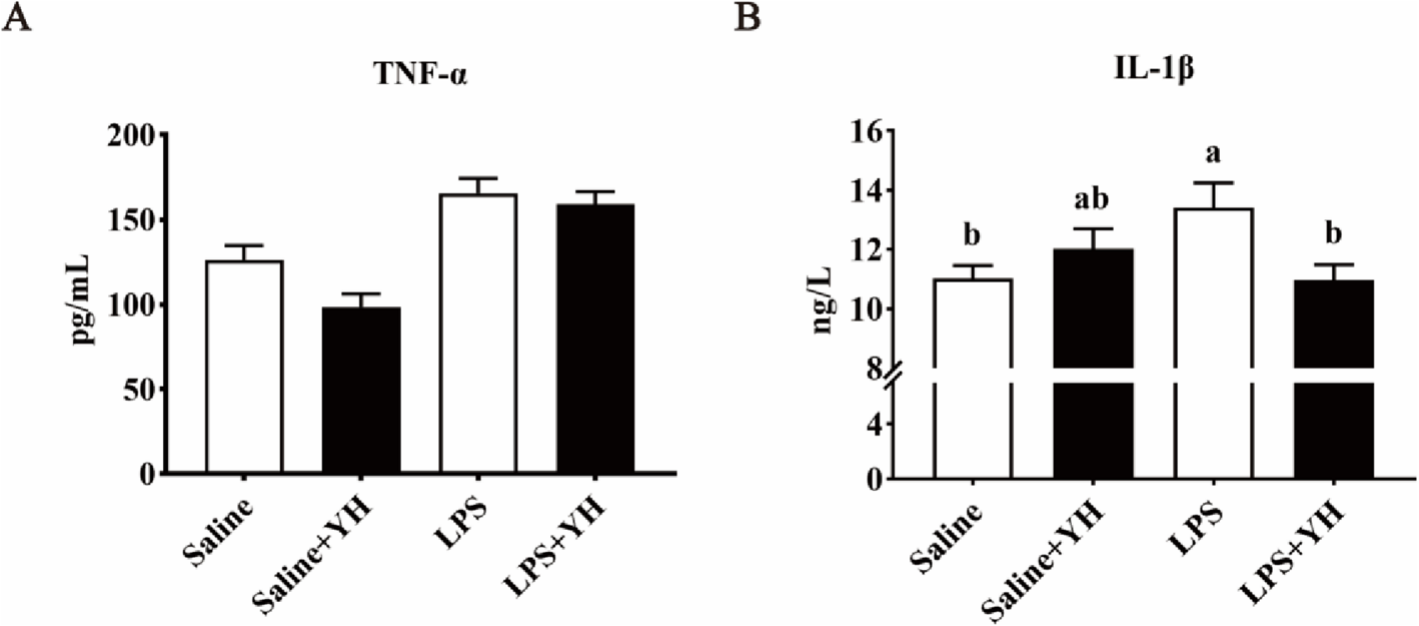
Dietary yeast hydrolysate supplementation attenuated the enhancement of serum concentrations of (**A)** tumor necrosis factor-α (TNF-α) and (**B)** interleukin-1β (IL-1β) in piglets challenged with LPS. Control, piglets were fed with a basal diet; YH, piglets were fed with a YH containing diet, 5 g/kg. ^a,^^b^Means with different superscript letters in a row were significantly different (*P* < 0.05)

Results of serum acute phase protein, stress hormone and inflammatory cytokines concentrations in piglets challenged with LPS were showed in Fig. 2 and Fig. 3. As expected, LPS injection enhanced HP (Fig. 2B), cortisol (Fig. 2D), ACTH (Fig. 2E) and IL-1β (Fig. 3B) concentrations in serum (*P* < 0.01). However, LPS + YH group significantly decreased the concentrations of HP, cortisol, ACTH and IL-1β in serum compared with the LPS group (*P* < 0.05).

**Fig. 4:**
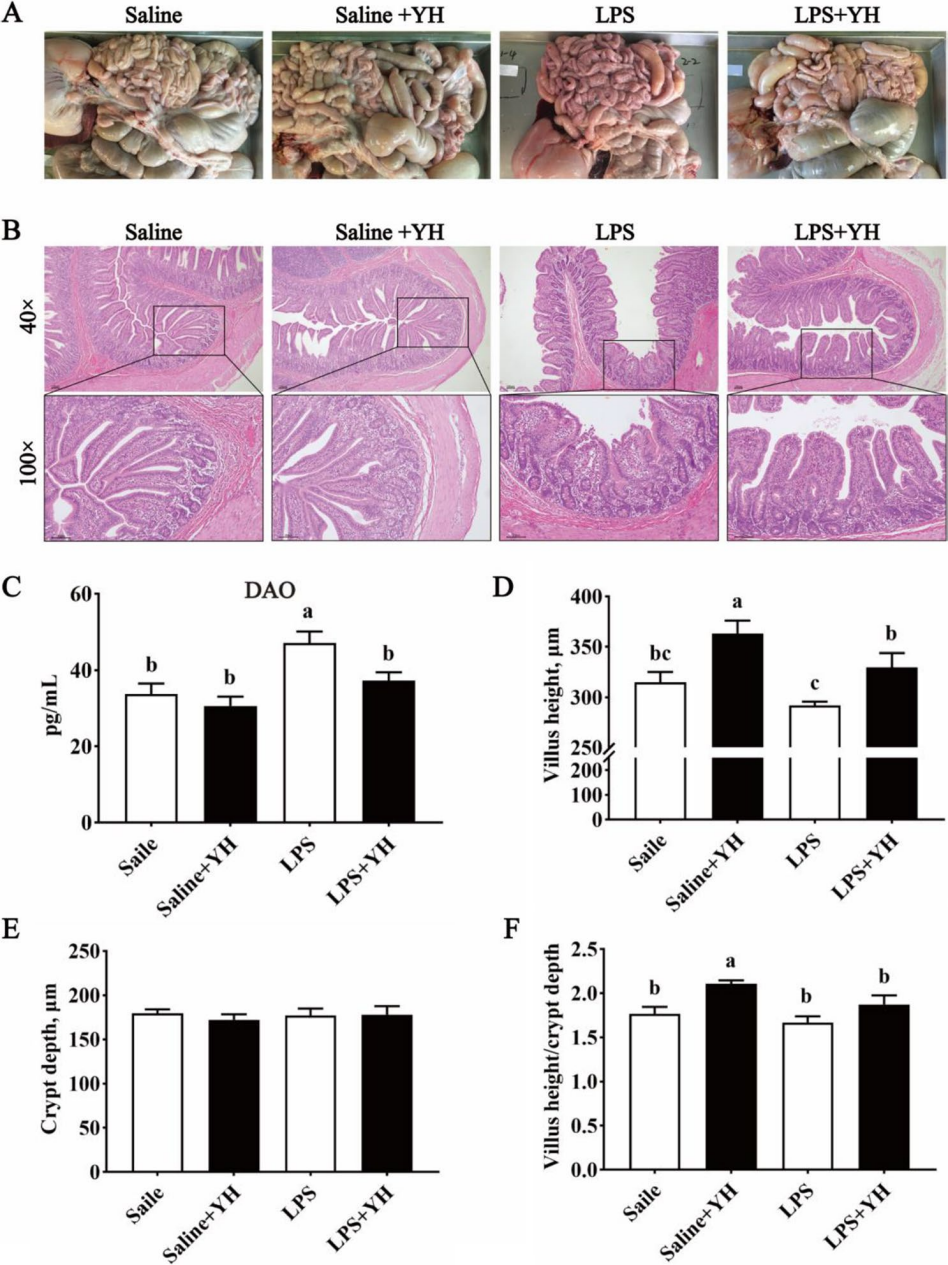
Dietary yeast hydrolysate supplementation attenuated the effects of LPS-injection on jejunal permeability and morphology. **A** Representative picture of the appearance of the intestinal tract of a piglet. **B** Hematoxylin and eosin section of jejunum at 40 times magnification (up) and 100 times magnification (down). **C** The concentrations of diamine oxidase (DAO) in serum of piglets. **D–F** The villus height, crypt depth and the villus height:crypt depth ratio of jejunum of piglets. Control, piglets were fed with a basal diet; YH, piglets were fed with a YH containing diet, 5 g/kg. ^a^^,^^b^^,^^c^Means with different superscript letters in a row were significantly different (*P* < 0.05)

As shown in Fig. 4, compared with saline group, there was greater concentration of serum DAO in LPS group (*P* < 0.01), however, YH supplementation significantly inhibited the increase of the serum DAO concentration in LPS challenged piglets (*P* = 0.02).

### Effects of YH on intestinal morphology in LPS- challenged piglets

The effects of YH on intestinal morphology in LPS-challenged piglets were shown in Fig. 4. LPS challenge caused dramatic intestinal hyperemia (Fig. 4A), induced intestinal mucosal injury reflected by villous atrophy and mucosal detachment (Fig. 4B), and decreased the villus height (Fig. 4D) (*P* = 0.02). Compared with LPS group, LPS + YH group attenuated the state of intestinal hyperemia, improved the morphology and significantly increased the villus height (*P* = 0.03).

### Effects of YH on the expression of intestinal barrier related genes in LPS-challenged piglets

**Fig. 5:**
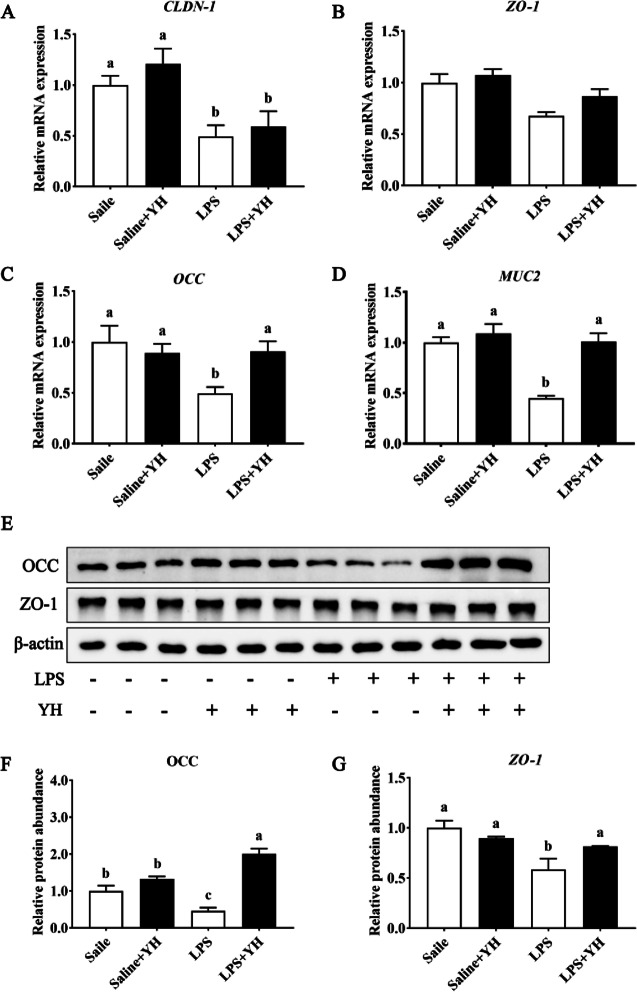
Dietary yeast hydrolysate supplementation improved the barrier function of jejunal mucosa in piglets challenged with LPS. **A–D** The relative mRNA expression of Claudin-1 (*CLDN-1*), Zonula occludens-1 (*ZO-1*), Occludin (*OCC*) and mucin2** (***MUC2*) in the jejunal mucosa of piglets. **E–G** The protein abundance of OCC ZO-1 in the jejunal mucosa of piglets. Control, piglets were fed with a basal diet; YH, piglets were fed with a YH containing diet, 5 g/kg. ^a^^,^^b^^,^^c^means with different superscript letters in a row were significantly different (*P* < 0.05)

Compared with saline treatment, LPS injection reduced the relative mRNA expressions of *CLDN-1* (Fig. 5A), *OCC* (Fig. 5C) and *MUC2* (Fig. 5D) (*P* < 0.05), as well as the protein expressions of OCC (Fig. 5E and 5F; *P* = 0.01) and ZO-1 (Fig. 5E and G; *P* < 0.01) in jejunal mucosa. In compared to LPS group, YH supplementation significantly inhibited the down-regulation of mRNA expression of *OCC* (*P* = 0.02) and *MUC2* (*P* < 0.01) and protein abundances of OCC (*P* < 0.01) and ZO-1 (*P* = 0.04) in jejunal mucosa of LPS-challenged piglets.

### Effects of YH on the expression of TNF-α and IL-1β of jejunum in piglets challenged with LPS

**Fig. 6:**
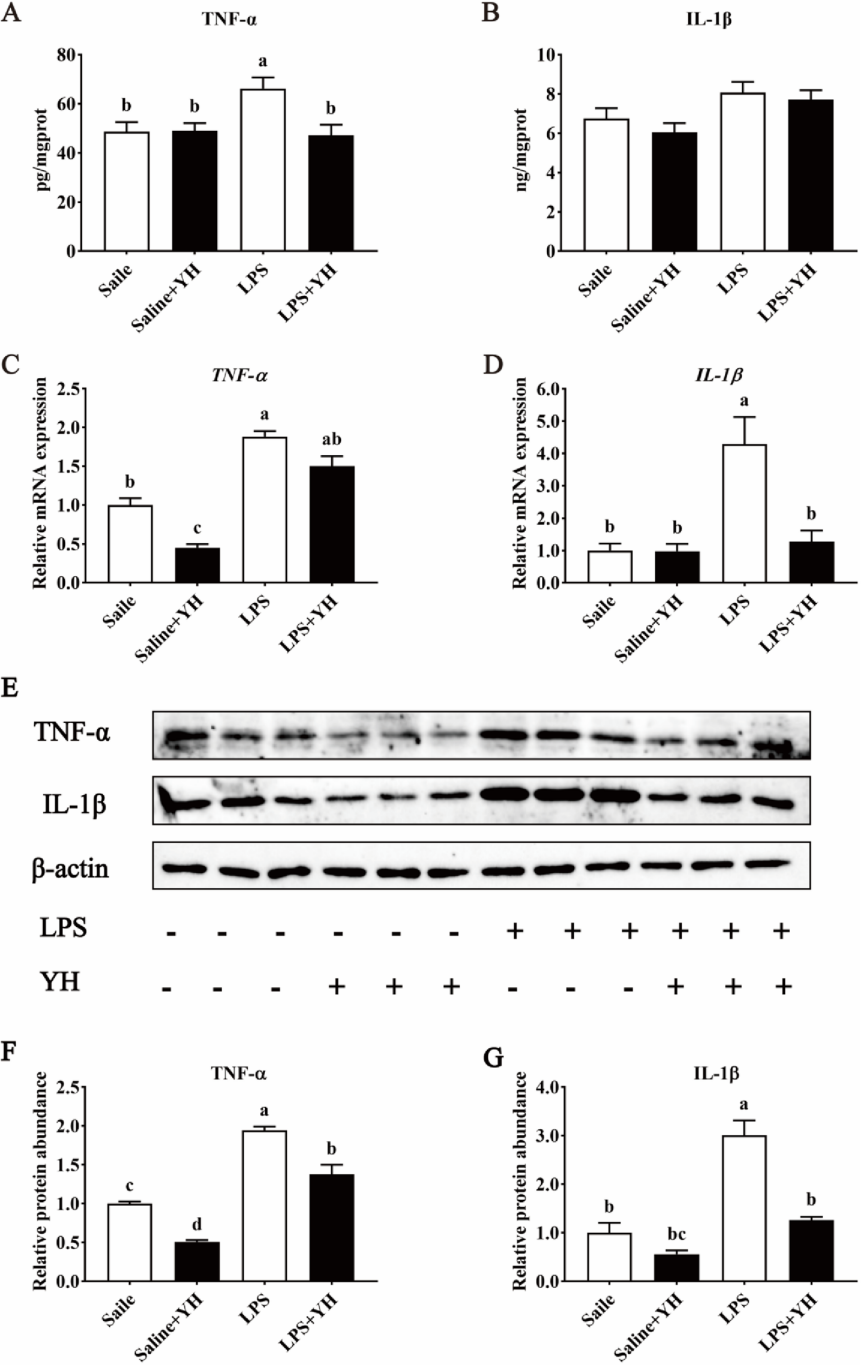
Dietary yeast hydrolysate supplementation inhibited the inflammatory response of jejunal mucosa in piglets challenged with LPS. **A** and** B** The concentrations of tumor necrosis factor-α (TNF-α) and interleukin-1β (IL-1β) in the jejunal mucosa of piglets by ELISA method. **C** and** D** The mRNA expression of *TNF-α* and *IL-1β* in the jejunal mucosa of piglets. **E–G** The protein abundance of TNF-α and IL-1β in the jejunal mucosa of piglets. Control, piglets were fed with a basal diet; YH, piglets were fed with a YH containing diet, 5 g/kg. ^a^^,^^b^^,^^c^Means with different superscript letters in a row were significantly different (*P* < 0.05)

As shown in Fig. 6, LPS injection enhanced the concentrations of TNF-α (Fig. 6A; *P* = 0.01) in jejunal mucosa, however, YH significantly reversed this change (*P* < 0.01). A further analysis by RT-qPCR and western blot revealed that LPS challenge significantly increased the mRNA expressions of *TNF-α* (Fig. 6C) and *IL-1β* (Fig. 6D) and the corresponding protein abundances (Fig. 6E–G; *P* < 0.01). Conversely, a down-regulation was observed in the mRNA expressions of *IL-1β* and the protein abundance of TNF-α and IL-1β in LPS + YH group (*P* < 0.01).

### Effects of YH on the TLR4/NF-κB signaling pathway in LPS-challenged piglets

**Fig. 7:**
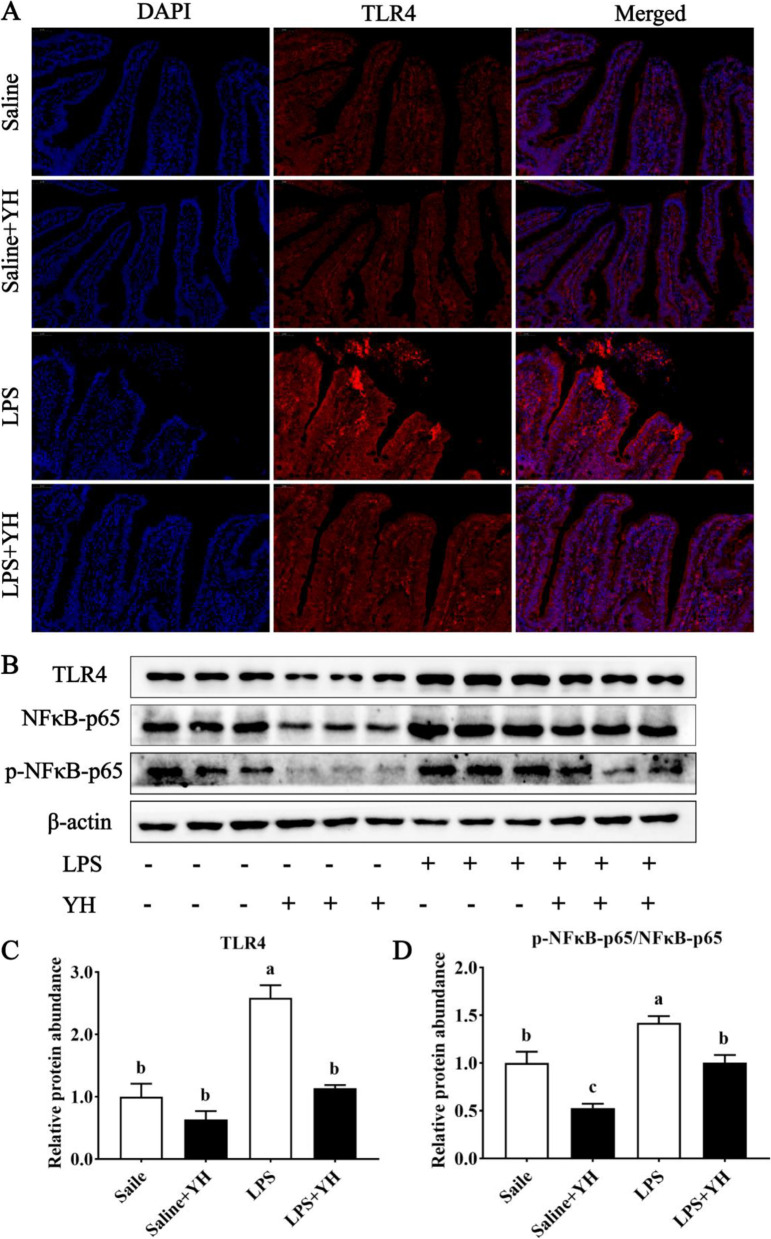
Dietary yeast hydrolysate supplementation decreased the protein abundance of TLR4 and p-NFκB-p65 in jejunal mucosa in piglets challenged with LPS. **A** Immunofluorescence staining (100 times magnification) of toll-like receptors 4 (TLR4) in jejunum of piglets. **B–D** Relative protein abundance of **B** and **C** TLR4 and **B** and** D** phosphor-Nuclear factor-κB-p65 (p-NFκB-p65). Control, piglets were fed with a basal diet; YH, piglets were fed with a YH containing diet, 5 g/kg. ^a^^,^^b^^,^^c^Means with different superscript letters in a row were significantly different (*P* < 0.05)

As shown in Fig. 7, we investigated the expression of TLR4, the major inflammation-associated receptor, by immunofluorescence analysis and discovered that the distribution of TLR4 in jejunum of piglets enhanced by LPS challenge (Fig. 7A). Nevertheless, the variability was reversed with YH supplementation when compared to LPS group. Moreover, we observed an up-regulation in the expressions of TLR4 protein (Fig. 7B and C) and p-NFκB-p65 protein (Fig. 7B and D) in LPS group compared with saline group (*P* < 0.01). LPS + YH group decreased the protein abundances of TLR4 (*P* < 0.01) and p-NFκB-p65 (*P* = 0.01) compared with LPS group.

## Discussion

Yeast hydrolysate (YH), an autolysate of *Saccharomyces cerevisiae*, has attracted much attention as a nutritional additive, which involved in various biological activities including growth promotion and immune regulation. Throughout the 21-d feeding experiment (pre-LPS-challenge) in this study, we observed that dietary YH supplementation increased growth performance of piglets. This effect may be related to the molecular weight of YH. As shown in an animal model, the low molecular weight YH (below 30 kDa) exhibits a low toxicity for rats [24]. This hydrolysate is more accessible to animals due to its low molecular weight contributing to a higher solubility in aqueous media and a better digestibility and absorptivity [36]. Previous studies have found that YH (below 10 kDa) revealed physiological effects on anti-obesity [37, 38] and anti-stress [20, 21]. In this study, the molecular weight of YH was less than 50 kDa and mostly clustered below 25 kDa. Furthermore, the beneficial effects of YH on growth performance have been characterized in rats [39], piglets [40], growing-finishing pigs [25], chickens [41] and fish [27, 42]. Similar results have been found in our previous study that feeding YH significantly improved growth performance for piglets under the normal physiological condition and the reason might also be that YH could improve intestinal barrier function [22]. Nevertheless, it remains to be studied whether YH can improve intestinal health under pathological states. In the present study, we focused on whether dietary YH attenuated the intestinal barrier impairment through inhibiting the massive release of pro-inflammatory cytokines. Hence, we utilized an incontrovertible model for acute gut injure by injecting lipopolysaccharide (LPS). LPS is an intrinsic constituent of membranes in gram-negative bacteria and it is a powerful endotoxin [7]. It binds to TLR4 and subsequently activates downstream signaling pathways, triggering an inflammatory response [11].

Acute phase proteins (APPs) such as C-reactive protein (CRP), haptoglobin (HP) and serum amyloid A (SAA) were secreted by hepatocytes and served as a crucial role in the etiopathogenesis of immune diseases [43]. In addition, some stress hormones (e.g., ACTH and cortisol) were frequently used in response to infectious status in the body [44]. With the infection and injury of the host (such as LPS injection), the expressions or serum concentrations of APPs and stress hormones will be dramatically enhanced [45, 46]. Indeed, our results found that serum concentrations of HP, SAA, ACTH and cortisol in piglets were increased 4 h after LPS-injection. However, supplementation of YH to the LPS-infected piglets reduced the levels of HP, ACTH and cortisol, suggesting that YH attenuated LPS-induced stress response. To our knowledge, TNF-α and IL-1β were the critical inducers of APPs [47]. And it is a highly significant correlation between serum APPs and TNF-α levels in some infectious agents [48]. In the present investigation, LPS challenge resulted in an increase of TNF-α and IL-1β in serum. Undoubtedly, LPS caused an excessive activation of immune system. Nevertheless, YH supplementation decreased the concentrations of TNF-α and IL-1β in LPS-challenged piglets, which indicated dietary YH supplementation could decrease the systemic inflammation after LPS infection. Over-production of proinflammatory cytokines manufactured intestinal damage, such as villous atrophy, mucosal swelling, submucosal hemorrhage and exfoliation, and led to an increase of intestinal permeability [7]. Several blood indictors have been used to evaluate the intestinal permeability. DAO, an enzyme found at high levels in the mammalian intestinal mucosa, is a marker of maturation and integrity in response to intestinal epithelium [49]. The levels of serum DAO are positively correlated with intestinal permeability. In this study, YH supplementation reduced DAO concentrations, indicating that YH had beneficial effects on attenuating the increase of intestinal permeability of piglets challenged with LPS. Meanwhile, intestinal permeability can usually be assessed with intestinal epithelial barrier function [6]. Therefore, it is possible that YH supplementation can mitigate systemic inflammatory associated with the improvement of intestinal barrier function.

Intestinal morphology is regarded as a visual reflection for the growth and development of gut and determined by villus height and crypt depth [50]. This study showed that LPS-injection induced intestinal damage including villous atrophy, mucosal detachment, and a decrease of villus height in jejunum of piglets. While dietary inclusion of YH could counteract the morphological changes of jejunum after LPS challenge, thereby maintaining the villus integrity and structure of intestinal mucosa. Considering the importance of intestinal epithelial barrier in intestinal function, the tight junctions between epithelial cells were analyzed in this study. Tight junctions are multi-protein complexes including claudins, occludin and ZOs, which defend against the passage of luminal antigens, pathogenic bacterium and their toxins [6]. Recently, the experimental results showed that YH inclusion markedly attenuated the down-regulation mRNA expressions of *OCC*, and the protein abundances of OCC and ZO-1 in jejunal mucosa of LPS-challenged piglets. Thus, dietary YH administration could maintain the integrity of intestinal barrier by inhibiting the decrease of tight junction protein expression under immunological stress, which might be the potential reasons that YH mitigated the impairments of intestinal permeability and morphology in LPS-injected piglets.

It is generally accepted that cytokines are the critical modulators of intestinal inflammation [47]. Over-production of pro-inflammatory cytokines (e.g., TNF-α and IL-1β) has been demonstrated to directly impair the tight junctional function of some epithelial and endocrine cells [51]. Previous studies have indicated that alleviating intestinal inflammation might effectively prevent pathogenic bacteria and their toxins from disrupting intestinal barrier function [2]. Consequently, the reduction of intestinal pro-inflammatory cytokine concentrations under stressful conditions is one of the major strategies to protect the intestinal barrier and mitigate intestinal inflammation. In the present study, YH supplementation significantly reduced the mRNA expressions of *TNF-α* and *IL-1β*, and the corresponding protein abundances in jejunal mucosa in LPS challenged-piglets. Similarly, Waititu et al. [52] reported that piglets receiving a YH riched in cell wall polysaccharides reduced TNF-α level in ileum challenged by LPS. Hence, the protected effect of YH on intestinal barrier appears to be achieved by suppressing the inflammatory response. This is also a possible reason that YH ameliorates the systemic inflammatory response.

With a further view to investigating the molecular mechanisms of YH in the alleviation of intestinal inflammation, we evaluated the activation of TLR4 signaling pathway. TLR4, a typical pattern recognition receptor in the TLR protein family, is widely distributed on the surface of various intestinal cells and plays an essential role in LPS-mediated signaling [11]. Mechanistically, TLR4 activation triggered by LPS can induce the increased expression of downstream molecules (such as NF-κB), and then enhance the expression of proinflammatory cytokines-related genes, resulting in intestinal barrier damage [7, 53]. Currently, we observed that YH supplementation significantly decreased the protein abundance and immunofluorescence intensity of TLR4 in jejunal mucosa of piglet challenged with LPS, as well as downregulated the protein levels of p-NF-κB. NF-κB, the master transcription factor for TLR4 signaling, is phosphorylated and translocated to the nucleus in the stimulated state, then promoting the release of pro-inflammatory cytokines [54]. Accordingly, YH supplementation inhibits the TLR4/NF-κB signaling pathway, which may represent a potential mechanism to decelerate the signaling of LPS, thereby enabling the inflammatory response to be dampened.

## Conclusion

Dietary YH supplementation improves the growth performance and attenuates LPS-induced intestinal inflammation and barrier injury. The underlying molecular mechanism is YH supplementation inhibits the activation of TLR4/NF-κB signaling pathway induced by LPS, to prevent the over-production of inflammatory cytokines, and thus improved the intestinal barrier function.

## Supplementary Information

**Additional file 1: Fig. S1.** SDS-PAGE analysis of yeast hydrolysate.

**Additional file 2: Table S1.** Chemical component of yeast hydrolysate.

## Abbreviations

ACTH: Adrenocorticotropic hormone
ADFI: Average daily feed intake
ADG: Average daily gain
CLDN-1: Claudin-1
CRP: C-reactive protein
DAO: Diamine oxidase
F/G: The ratio of feed intake to gain
HP: Haptoglobin
IL-1β: Interleukin-1β
LPS: Lipopolysaccharide
MUC2: Mucin2
NF-κB: Nuclear factor-κB
OCC: Occludin
SAA: Serum amyloid A
TLR4: Toll-like receptors 4
TNF-α: Tumor necrosis factor-α
YH: Yeast hydrolysate
ZO-1: Zonula occludens-1
