# Supplementary figures and images for: Yeast hydrolysate attenuates lipopolysaccharide-induced inflammatory responses and intestinal barrier damage in weaned piglets
Source: J Anim Sci Biotechnol. 2023 Mar 17;14:44. doi: 10.1186/s40104-023-00835-2 (PMC10021991; doi:10.1186/s40104-023-00835-2)

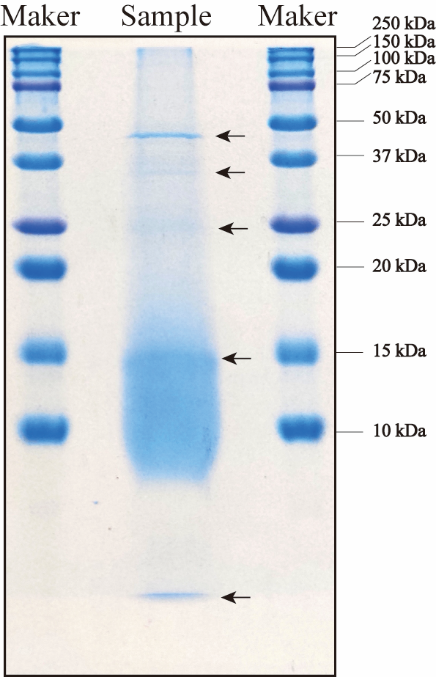


**Fig. S1** SDS-PAGE analysis of yeast hydrolysate

Supplement: Supplementary file 1 — Additional file 1: Fig. S1. SDS-PAGE analysis of yeast hydrolysate. [file 40104_2023_835_MOESM1_ESM.docx]
