# Supplementary material for: Yeast hydrolysate attenuates lipopolysaccharide-induced inflammatory responses and intestinal barrier damage in weaned piglets
Source: J Anim Sci Biotechnol. 2023 Mar 17;14:44. doi: 10.1186/s40104-023-00835-2 (PMC10021991; doi:10.1186/s40104-023-00835-2)
Supplement: Supplementary file 2 — Additional file 2: Table S1. Chemical component of yeast hydrolysate. [file 40104_2023_835_MOESM2_ESM.docx]

**Table S1** Chemical component of yeast hydrolysate

| **Composition** | **Moisture,**  **%** | **Crude protein,**  **%** | **Crude fat,**  **%** | **Crude ash,**  **%** | **Gross energy, cal/g** |
| --- | --- | --- | --- | --- | --- |
| Content | 3.95 | 45.50 | 2.17 | 6.47 | 3825.00 |
